# Protein Degradation of RNA Polymerase II-Association Factor 1(PAF1) Is Controlled by CNOT4 and 26S Proteasome

**DOI:** 10.1371/journal.pone.0125599

**Published:** 2015-05-01

**Authors:** Hwa-Young Sun, Nari Kim, Cheol-Sang Hwang, Joo-Yeon Yoo

**Affiliations:** Department of Life Sciences, Pohang University of Science and Technology, Pohang, Korea; Pohang University of Science and Technology (POSTECH), KOREA, REPUBLIC OF

## Abstract

The PAF complex (PAFc) participates in various steps of the transcriptional process, from initiation to termination, by interacting with and recruiting various proteins to the proper locus for each step. PAFc is an evolutionarily conserved, multi-protein complex comprising PAF1, CDC73, CTR9, LEO1, yRTF1 and, in humans, hSKI8. These components of PAFc work together, and their protein levels are closely interrelated. In the present study, we investigated the mechanism of PAF1 protein degradation. We found that PAF1 protein levels are negatively regulated by the expression of CNOT4, an ortholog of yNOT4 and a member of the CCR4-NOT complex. CNOT4 specifically controls PAF1 but not other components of PAFc at the protein level by regulating the polyubiquitination of PAF1 and its subsequent degradation by the 26S proteasome. The degradation of PAF1 was found to require nuclear localization, as no PAF1 degradation by CNOT4 and the 26S proteasome was observed with NLS (nucleus localization signal)-deficient PAF1 mutants. However, chromatin binding by PAF1 was not necessary for 26S proteasome- or CNOT4-mediated degradation. Our results suggest that CNOT4 controls the degradation of chromatin-unbound PAF1 via the 26S proteasome.

## Introduction

Transcription is a highly complicated biochemical process which RNA is synthesized from a DNA template by RNA polymerase. To properly respond and adapt to internal and external environmental changes, the production, assembly, sub-cellular localization, and degradation of transcription-associated complexes are tightly controlled [1, 2]. In addition, post-translational modifications of histones or the transcriptional machinery can specifically adjust the association of these molecules with RNA polymerase in the chromatin [3, 4].

CCR4-NOT is an evolutionarily conserved multi-functional protein complex that functions in many aspects of transcriptional regulation, primarily controlling mRNA and protein life cycles from synthesis to degradation [5, 6]. In yeast, the CCR4-CAF1 module of the CCR4-NOT complex controls mRNA stability via its deadenylase enzymatic activity, whereas the NOT2-3/5 module associates with the transcriptional machinery. In addition, the NOT4 E3 ligase module controls the protein stability of transcription factors via RING domain-mediated ubiquitination [7]. The CCR4-NOT complex also interacts with proteasomal subunits to control the assembly of the proteasome complex [8].

The CCR4-NOT complex additionally functions to control the global and locus-specific methylation status of histones. The yNOT4 E3 ligase ubiquitinates and destabilizes the histone H3 Lys4 (H3K4) demethylase yJHD2 to maintain appropriate cellular levels of H3K4 trimethylation [9]. As a result, the loss of yNOT4 expression correlates with a global reduction of H3K4me3 but not H3K4me1/2, H3K36me2, or H3K79me2/3 levels. yNOT4 has also been reported to alter H3K4me3 levels in a locus-specific manner by controlling the recruitment of the PAF complex (PAFc) [10]. However, the detailed molecular mechanism underlying the regulation of PAFc by NOT4 remains to be elucidated.

PAFc is an evolutionarily conserved transcriptional complex that associates with RNA polymerase II (Pol II). It is composed with PAF1, CDC73, CTR9, LEO1, RTF1 in yeast and contains an additional component SKI8 in human [11, 12]. PAFc participates in histone modification and transcriptional processes ranging from initiation to termination and typically functions via protein-protein interactions with histone modifiers, elongation factors, and RNA 3’-end processing factors [13–15]. Through transcriptional regulation, PAFc is involved in various cellular processes including cell cycle, development, immune response and tumor progression. The hPAF1 overexpression to NIH3T3 induces cell growth and tumor formation [16]. Abnormal PAFc expression is correlated with diverse types of cancers, including the pancreatic cancers and parathyroid tumors [17].

The functional integrity and structural stability of the PAFc multi-protein complex is sensitive to the stoichiometry of its individual components. yCDC73 or yRTF1 deficiency results in the dissociation of PAFc from RNA Pol II and chromatin [13, 18], and in human cells, hPAF1-deficient hPAFc fails to interact with RNA Pol II [14]. Furthermore, the depletion of one PAFc subunit has been shown to result in the reduction of other components in multiple species, from yeast to humans, suggesting that the overall stability of PAFc is sensitive to the stability of its individual components [19–21]. Although the level of PAFc protein is tightly linked to the complex formation, the mechanisms by which the protein stabilities of the individual PAFc components are regulated remain unclear.

In this report, we demonstrate that the protein level of PAF1 is controlled by CNOT4, a mammalian ortholog of NOT4. PAF1 is K48 polyubiquitinated *in vivo* in a CNOT4-dependent manner and is degraded by the 26S proteasome.

## Materials and Methods

### Cell culture and reagents

HEK293 or HepG2 cells (ATCC) were cultured in DMEM or MEM (Welgene), respectively, with 10% fetal bovine serum (Hyclone). Transient transfection of siRNA and mammalian expression plasmids were performed with Lipofectamine 2000 (Invitrogen). To block protein degradation, cells were treated with MG132, chloroquine (CQ), NH_4_Cl (Sigma-Aldrich) or PS341 (Biovision) for the indicated times.

### Plasmids and siRNAs

The Myc-mPAF1 plasmid has been described previously [22]. For the CNOT4 expression vector, CNOT4 CDS was obtained by PCR using cDNA from HEK293 cells and cloned into the pEF-DEST plasmid (Invitrogen). The RING domain deletion mutant of CNOT4 was generated by site-directed mutagenesis. Control or CNOT4 siRNAs were purchased from GenePharma. The oligomer sequences for each siRNA were as follows: control (5’-UUCUCCGAACGUGUCACGUTT), CNOT4 #1 (5’-GGCGGGUAAACACCAAGAATT), CNOT4 #2 (5’-GAUUUACUACAGAGUUCAATT), CNOT4 #3 (5’-AGUCGUUAUUCUC AGACAATT), CNOT4-UTR (5’-GCAGUCUCAAUACUUAAATT).

### RNA preparation and analysis

Total RNA was isolated from cells using RNAiso Plus (TAKARA Bio). Total RNA (1 μg) was used for reverse transcription with an oligo-d(T)15 primer (Promega). The synthesized cDNA was used for conventional or quantitative real-time PCR. The sequences of the primers used for PCR were as follows: *Actin*, 5’-CATGTACGTTGCTATCCAGGC-3’ and 5’-CTCCTTAATGTCACGCACGAT-3’; *PAF1*, 5’-GATGATGAGGACAGAGGACA-3’ and 5’-CAGTCACTGTCACTATCAGC-3’; *CNOT4*, 5’-TATCCAGAAGACCCAGCAGT-3’ and 5’-ATAAGCACTGGCACTTGGAC-3’.

### Immunoblotting and fractionation

Cell lysates or chromatin-unbound and bound fractions were prepared and separated as described previously [22, 23]. Antibodies against PAF1, LEO1, CDC73 and CTR9 were purchased from Bethyl Laboratories (Montgomery). Anti-LAMIN B, ACTIN, CYCLIN A, c-Myc, PARP1, GFP and H3 antibodies were purchased from Santa Cruz Biotechnology, anti-CNOT4 from Abcam, anti-GAPDH from Chemicon, anti-HA from Roche and anti-K48-UB from Millipore.

### Immunofluorescent staining

Experimental procedure was followed as described previously [22]. Samples were analyzed with an epifluorescence microscope (Zeiss) or FV1000 confocal microscope (Olympus).

### In vivo ubiquitination assay

Cells were lysed in 1% SDS-containing lysis buffer (25 mM Tris-HCl pH 7.5, 150 mM NaCl, 0.5% NP-40, 1% SDS) with protease inhibitors and 40 mM NEM at 95°C for 10 min. After centrifugation at 13000 rpm at 4°C for 30 min, the supernatants were collected and mixed with nine volumes of lysis buffer (25 mM Tris-HCl pH 7.5, 150 mM NaCl, 0.5% NP-40, protease inhibitors and 40 mM NEM) without SDS. The prepared cell lysates were pre-cleared with mouse IgG and protein G plus protein A agarose beads (Calbiochem) at 4°C for 1 hr, followed by incubation with an anti-cMyc antibody at 4°C for 6 hr. Protein G plus protein A agarose beads were added for an additional 1.5 hr. Beads were washed with 0.1% SDS-containing lysis buffer three times, and the protein eluent was analyzed by immunoblotting.

### Immunoprecipitation

Experimental procedure was followed as described with some modification [22]. Cells were lysed in interaction buffer (25mM Tris-HCl, 150mM NaCl, 0.5% NP-50) with protease inhibitors.

### Pulse-chase experiment

Cells were incubated in DMEM (+10% FBS) devoid of methionine (Met) for 1 hr at 37°C, then labeled with ^35^S-Met (PerkinElmer) for 1 hr. After pulse labeling, cells were washed with PBS and cultured in complete DMEM for the indicated times to chase. Metabolically labeled V5-mPAF1 was harvested by immunoprecipitation and analyzed by autoradiography.

## Results

### The steady-state protein level of PAF1 is controlled by CNOT4

**Fig 1:**
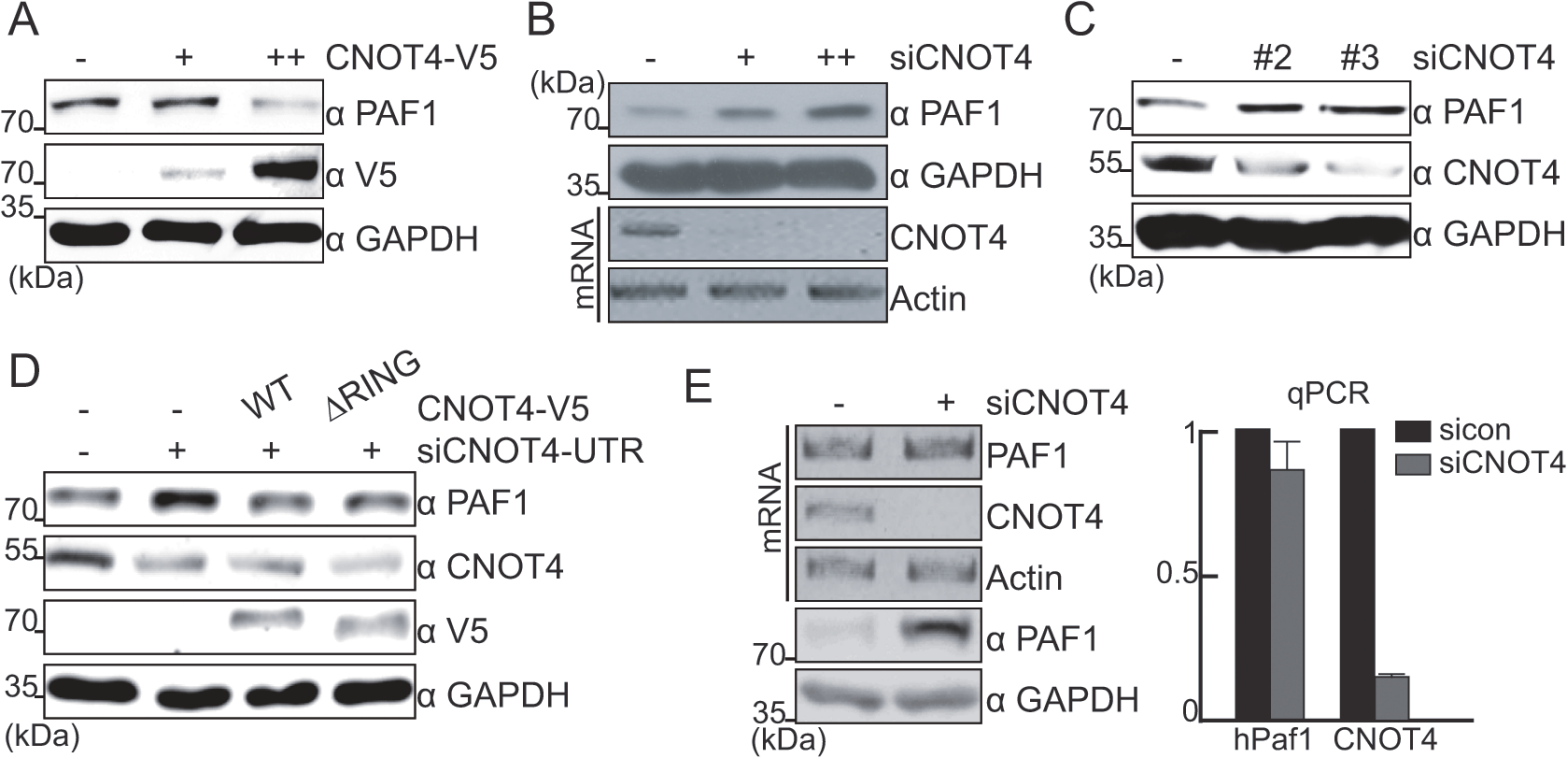
PAF1 is regulated by CNOT4 at the protein level. (A, B) The levels of endogenous PAF1 protein in CNOT4-overexpressing or silenced whole-cell lysates were analyzed by immunoblot. The silencing efficiency of CNOT4 was measured by RT-PCR (B). (C) The PAF1 and CNOT4 protein levels were measured in cells transfected with different siRNAs. (D) HEK293 cells were transfected with control or CNOT4 siRNA specific to the UTR, along with CNOT4 expression vectors. The protein levels of endogenous PAF1, endogenous CNOT4 and overexpressed V5-CNOT4 were measured by immunoblot assay. (E) After CNOT4 siRNA was transfected into HEK293 cells, PAF1 and CNOT4 mRNA levels were measured by conventional PCR (left) and quantitative real-time PCR (right). The protein levels of endogenous PAF1 were detected by immunoblot assay (left, bottom).

Because the recruitment of PAFc to chromatin is impaired in Not4-defecient *S*. *cerevisiae* [10], we first examined the potential regulatory effect of the CCR4-NOT complex on the PAF1 protein. We transiently overexpressed or silenced CNOT4, an ortholog of yNOT4, in HEK293 cell and measured the steady-state protein levels of PAF1. CNOT4 overexpression resulted in the disappearance of PAF1 (Fig 1A), whereas the depletion of CNOT4 expression led to the increased PAF1 levels (Fig 1B). To avoid any off-target effects, we used various siCNOT4s that each targeted the ORF or 3’UTR region of CNOT4. In each case, CNOT4 silencing consistently increased PAF1 protein levels (Fig 1C and 1D). To confirm the CNOT4 effect on PAF1, we performed rescue experiments using an siRNA targeting the CNOT4 3’UTR. The effect of CNOT4 silencing on PAF1 expression could be successfully reversed by the overexpression of full-length CNOT4-V5, indicating that the steady-state protein levels of PAF1 were controlled by CNOT4 (Fig 1D). The RING domain of yNOT4 was previously reported to be responsible for its E3 ligase enzymatic activity and was required to mediate the stability of target proteins [9]; therefore, we examined the contribution of the RING domain of CNOT4 using the CNOT4 (ΔRING) mutant. Contrary to our expectations, however, the overexpression of the CNOT4 (ΔRING) mutant successfully rescued the silencing effect of CNOT4 siRNA, similarly to that of WT CNOT4 (Fig 1D), indicating that the PAF1 protein levels are regulated by CNOT4 in a RING-domain-independent manner.

Because the CCR4-NOT complex controls mRNA stability [5], PAF1 mRNA levels were separately measured in CNOT4-silenced HEK293 cells. Although endogenous PAF1 protein was observed to accumulate in response to CNOT4 silencing, PAF1 mRNA levels were not significantly affected (Fig 1E). Taken together, these results suggest that CNOT4 regulates PAF1 at the protein level.

### The level of PAF1 protein, but not other PAFc components, was controlled by CNOT4

**Fig 2:**
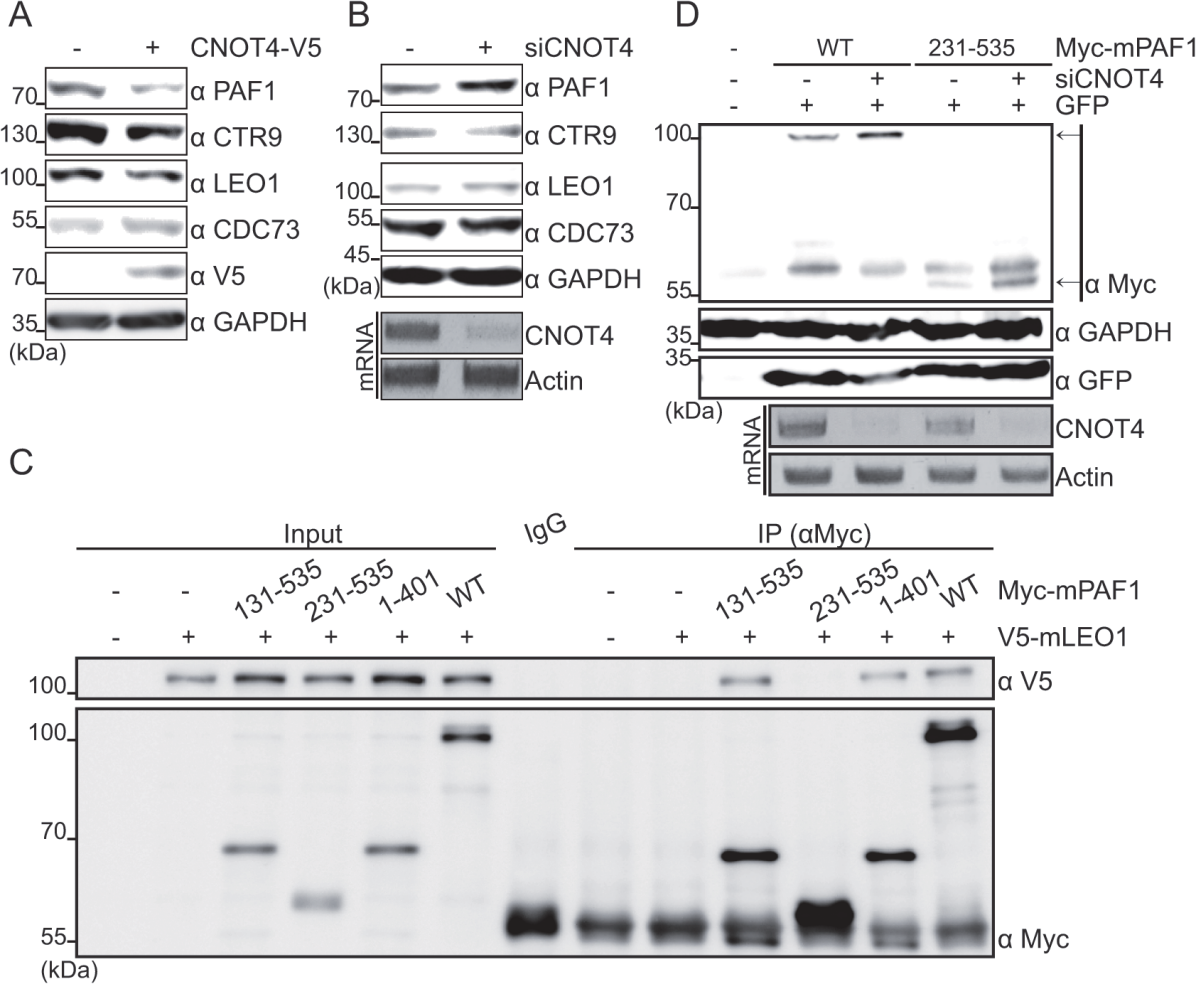
CNOT4 regulates PAF1 but not other PAFc components. (A, B) Endogenous PAFc protein levels were measured in CNOT4-overexpressing or silenced HEK293 cells via Western blot. The silencing efficiency of CNOT4 was measured by RT-PCR (B, bottom). (C) Cells were transfected with the indicated plasmids, and cell lysates were prepared and immunoprecipitated with Anti-Myc. The interaction between mPAF1 and mLEO1 was determined by Western blotting. (D) Myc-mPAF1 WT or 231–535 was transfected along with sicontrol or siCNOT4 in HEK293 cells. Myc-tagged mPAF1 proteins levels were analyzed by immunoblotting, and CNOT4 depletion was measured by RT-PCR (bottom).The arrows indicate the Myc-mPAF1 of WT or mutant.

Next, we examined whether CNOT4 affects the protein levels of other PAFc components. CNOT4 overexpression led to reductions in CTR9 and LEO1, as well as PAF1 (Fig 2A). However, as the depletion of one PAFc component has been reported to reduce the levels of other PAFc components [19–21], it was therefore unclear whether CNOT4 controls all of the affected PAFc proteins or selectively targets only one protein. Thus, we next silenced endogenous CNOT4 expression using siRNA and looked for alterations in the endogenous protein levels of other PAFc components. Knocking down CNOT4 expression led to increased levels of PAF1, but not other PAFc components, suggesting that CNOT4 targets only PAF1 (Fig 2B).

Newly synthesized PAF1 can exist either as a free protein or as part of the PAFc complex. To investigate whether CNOT4 targets PAF1 in its free form or within PAFc, we used the mPAF1 231–535 mutant, which fails to interact with the PAFc component LEO1 (Fig 2C). As observed with WT mPAF1, the protein levels of mPAF1 231–535 were similarly increased in the CNOT4-silenced cells (Fig 2D). Therefore, the regulation of PAF1 protein levels by CNOT4 does not require interactions between PAF1 and other PAFc components.

### CNOT4 regulates the 26S proteasome-mediated degradation of PAF1

**Fig 3:**
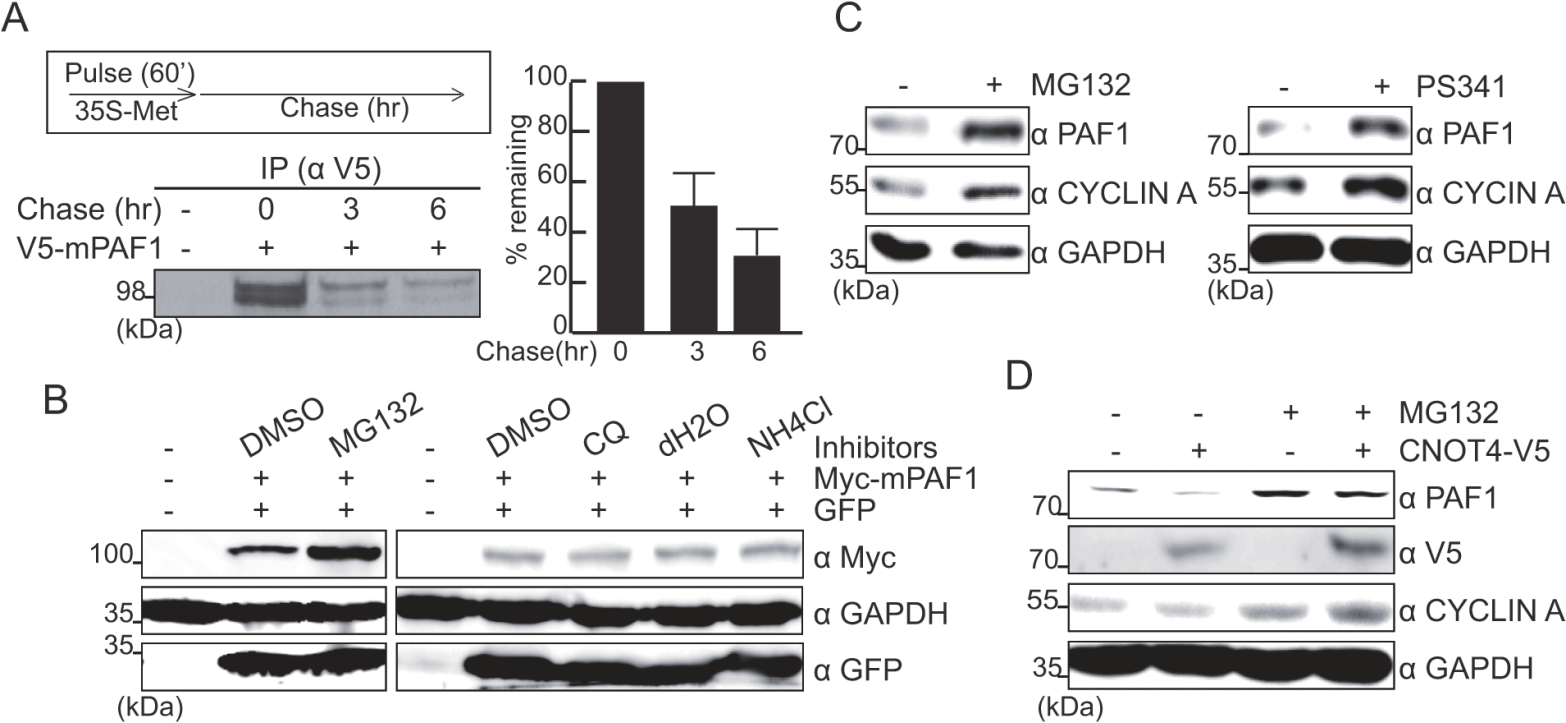
CNOT4 controls the degradation of PAF1 via the 26S proteasome. **(**A) Pulse-chase experiment. V5-mPAF1-transfected cells were metabolically labeled with ^35^S-Met for 60 min and chased for the indicated time periods. V5-mPAF1 was immunoprecipitated with anti-V5, and the remaining V5-mPAF1 protein was analyzed by autoradiography (left, bottom) and quantified (right). **(**B, C). Myc-mPAF1-transfected (B) or non-transfected (C) HEK293 cells were treated with the indicated inhibitors for 6 hr: MG132 (10 μM), chloroquine (CQ, 50 μM), NH_4_Cl (20 mM), PS341 (10 μM). Exogenous Myc-mPAF1 (B) or endogenous PAF1 (C) proteins in whole-cell lysates were detected with anti-Myc or anti-PAF1 antibodies, respectively. **(**D) Empty or CNOT4-V5-transfected cells were treated with MG132 (10 μM) for 6 hr. Total cellular extracts were prepared, and each protein was detected by immunoblot assay.

The protein half-life of PAF1 was estimated to be approximately 3 hr (Fig 3A). Compared to the average turnover rate of mammalian cellular proteins, PAF1 can thus be classified as a relatively unstable protein [24]. Because most cellular proteins are degraded via 26S proteasome- or lysosome-dependent pathways, HEK293 cells were treated with specific inhibitors of each degradation pathway to identify the specific pathway responsible for PAF1 protein degradation (Fig 3B and 3C). When 26S proteasome activity was inhibited by MG132, the cellular levels of exogenous Myc-tagged PAF1 (Myc-mPAF1) were increased. However, lysosomal inhibitors such as CQ or NH_4_Cl failed to alter Myc-mPAF1 protein levels (Fig 3B). Endogenous PAF1 protein levels were similarly increased by treatment with MG132 or with another inhibitor of the 26S proteasome, PS341 (Fig 3C). Because we observed that CNOT4 controls steady-state PAF1 protein levels, we next examined whether CNOT4 affects the 26S proteasomal degradation of PAF1. CNOT4-V5-transfected HEK293 cells were treated with either DMSO or MG132, and steady-state levels of endogenous PAF1 protein were determined (Fig 3D). MG132 treatment eliminated the suppressive effects of CNOT4 on PAF1 protein levels, indicating that CNOT4 controls the PAF1 protein via 26S proteasome-dependent degradation.

### CNOT4 affects the ubiquitination status of PAF1

**Fig 4:**
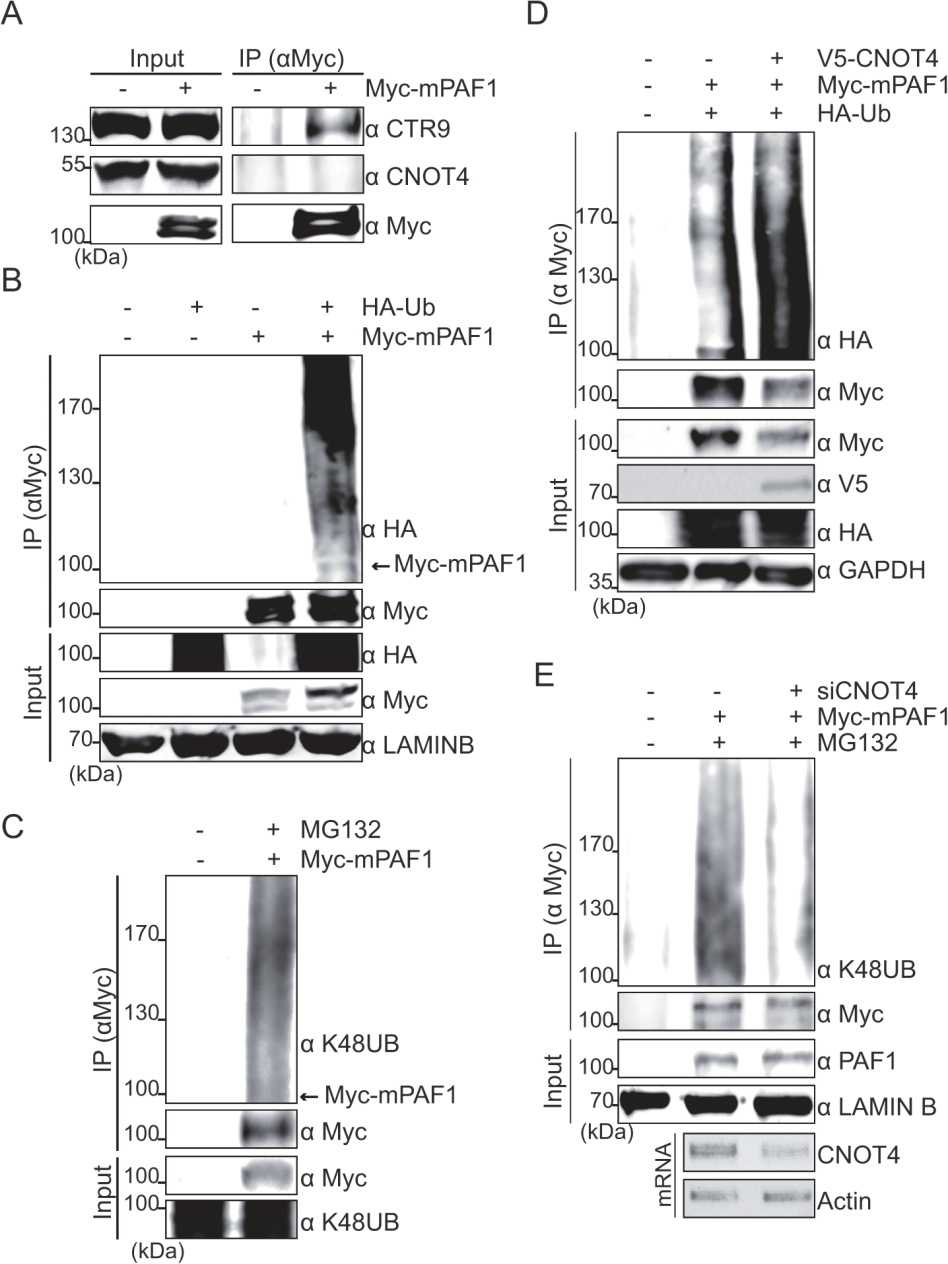
CNOT4 regulates the ubiquitination status of the PAF1 protein. (A) Physical interaction between PAF1 and CNOT4. Myc-mPAF1 was overexpressed in HEK293 cells. Myc-mPAF1 was immunoprecipitated from cell lysates, and the physical interaction between PAF1 and CNOT4 was assessed via immunoblotting. CTR9 was used as the positive control. **(**B) The indicated plasmids were transfected into HEK293 cells. After 48 hr, cells were harvested, and cell extracts were subjected to immunoprecipitation with an anti-Myc antibody. Ubiquitination of mPAF1 was detected by immunoblot assay with anti-HA. **(**C) Myc-mPAF1-transfected cells were treated with MG132 (10 μM) for 6 hr before harvest. Myc-mPAF1 was immunoprecipitated using an anti-Myc antibody and blotted with anti-Myc or anti-K48UB antibodies. (D) HEK293 cells were transfected with the indicated plasmids. Myc-mPAF1 was immunoprecipitated with anti-Myc from whole-cell lysates and blotted with anti- HA or anti-Myc antibodies. (E) HEK293 cells were transfected with Myc-mPAF1 along with control or CNOT4 siRNA. After treatment with MG132 (10 μM) for 6 hr, whole-cell extracts were immunoprecipitated with an anti-Myc antibody. Immunoprecipitates were subjected to Western blot analysis using anti-Myc or anti-HA antibodies. As the input control, 5% of the total lysates used for immunoprecipitation was loaded. The knockdown efficiency of siCNOT4 was measured by RT-PCR.

Although we demonstrated that CNOT4 controls the 26S proteasome-mediated degradation of PAF1 protein, the E3 ligase activity of CNOT4 was not required for this process (Fig 1D). There was also no obvious interaction between CNOT4 and PAF1 (Fig 4A), indicating that CNOT4 does not directly control PAF1 degradation. To further elucidate the role of CNOT4 in PAF1 degradation, we next examined whether the ubiquitination status of PAF1 was affected by CNOT4. Most proteins that are degraded via the 26S proteasome are marked by K48-linked poly-ubiquitination [25, 26]; the PAF1 protein was found to be poly-ubiquitinated under basal conditions, and the K48-linked poly-ubiquitination of PAF1 was easily detected in MG132-treated cells (Fig 4B and 4C). When CNOT4 was overexpressed, the levels of poly-ubiquitinated PAF1 protein were increased, whereas the K48-linked poly-ubiquitination of PAF1 was dramatically reduced in CNOT4-silenced cells (Fig 4D and 4E). Therefore, these results indicate that CNOT4 dose not directly ubiquitinate PAF1 but functions either during or prior step of the ubiquitination of PAF1.

### PAF1 nuclear localization is required for its degradation by the 26S proteasome and CNOT4

**Fig 5:**
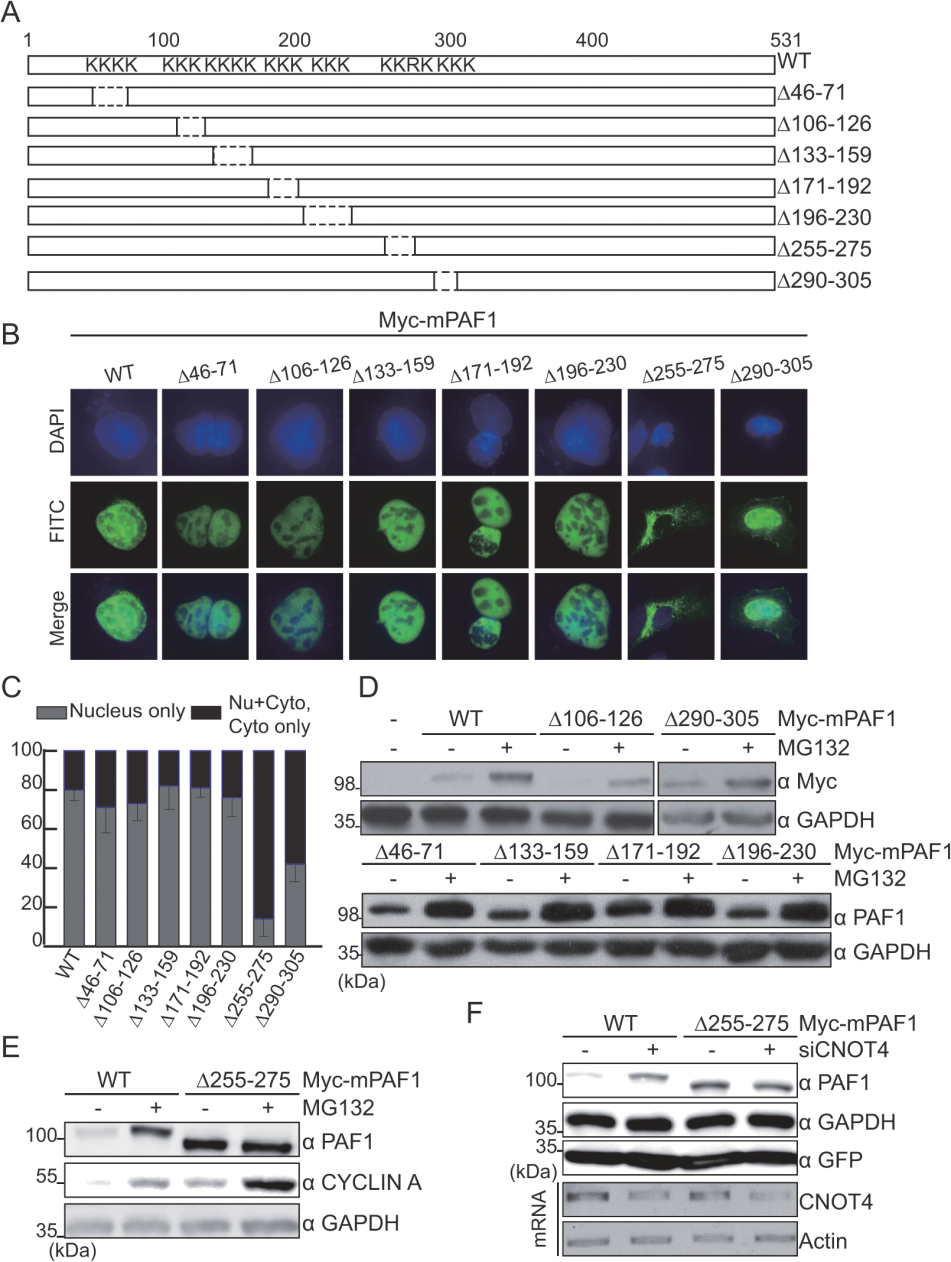
The 255–275 amino acid on PAF1 is required for its degradation. (A) Schematic diagram of WT and mutant PAF1. “K” designates lysine residues. **(**B, C) Cells were transfected with Myc-tagged WT or mutant PAF1, and the sub-cellular localization of these proteins was analyzed by immunofluorescence staining with an anti-Myc antibody (green) and DAPI (blue) (B). The percentages of cells containing WT or mutant PAF1 in the “nucleus only” or in the “nucleus and cytoplasm, cytoplasm only” are shown (C). (D) HEK293 cells were transfected with the indicated plasmids for 48 hours. Cells were treated with MG132 (10 μM) for 6 hr, followed by immunoblot analysis. **(**E) WT PAF1- or mPAF1△255-275-transfected cells were treated with MG132 (10 μM) for 6 hr, and the levels of each PAF1 protein were detected by immunoblot against anti-PAF1. **(**F) Cells were transfected with WT or mutant PAF1 along with control or CNOT4 siRNA. *Top*, WCLs were prepared from half of the cells and subjected to Western blot analysis. *Bottom*, Total RNA was prepared from the remaining cells and analyzed by RT-PCR.

The ubiquitin-26S proteasome machinery localized and functions in both nucleus and cytoplasm. Proteins degraded by 26S proteasome often requires specific signals permitting recognition by E3 ligase. Some proteins required the change of cellular localization. Others are degraded after DNA binding or post-translational modification, such as phosphorylation and ubiquitination [27–30]. Because PAF1 mainly localizes to and functions in the nucleus, the nuclear localization of PAF1 may affects its degradation. To test this hypothesis, we first generated a series of PAF1 deletion mutants and examined the sub-cellular localization patterns of wild-type (WT) and mutant PAF1 (Fig 5A). Most of the PAF1 deletion mutants exhibited sub-cellular localization patterns similar to that of WT PAF1, with the exceptions of PAF1△255–275 and PAF1△290–305 (Fig 5B and 5C). In particular, the PAF1△255–275 mutant, which includes the deletion of a canonical nuclear localization signal (NLS; KKRK), was primarily observed in the cytoplasm of transfected cells. A similar region was previously suggested to control the nuclear localization of PAF1 [16], leading us to conclude that the PAF1△255–275 mutant lacks an NLS.

WT or mutant PAF1-transfected cells were then treated with MG132, and changes in PAF1 protein levels were measured. In the case of WT PAF1, as well as the PAF1 mutants that normally localize to the nucleus, PAF1 protein was observed to accumulate in MG132-treated cells (Fig 5D). In contrast, the protein levels of the PAF1△255–275 mutant, which fails to enter the nucleus, were not altered by MG132 treatment (Fig 5E). In addition, the protein levels of the PAF1△255–275 mutant were not altered by CNOT4 silencing (Fig 5F). Although it is not clear whether the ubiquitination and nuclear localization motifs are inter-linked within the region spanning amino acids 255–275, these results nevertheless indicate that the nuclear localization of PAF1 is required for the 26S proteasome- and CNOT4-mediated degradation of PAF1.

### Chromatin association is not required for the controlled degradation of PAF1 protein

**Fig 6:**
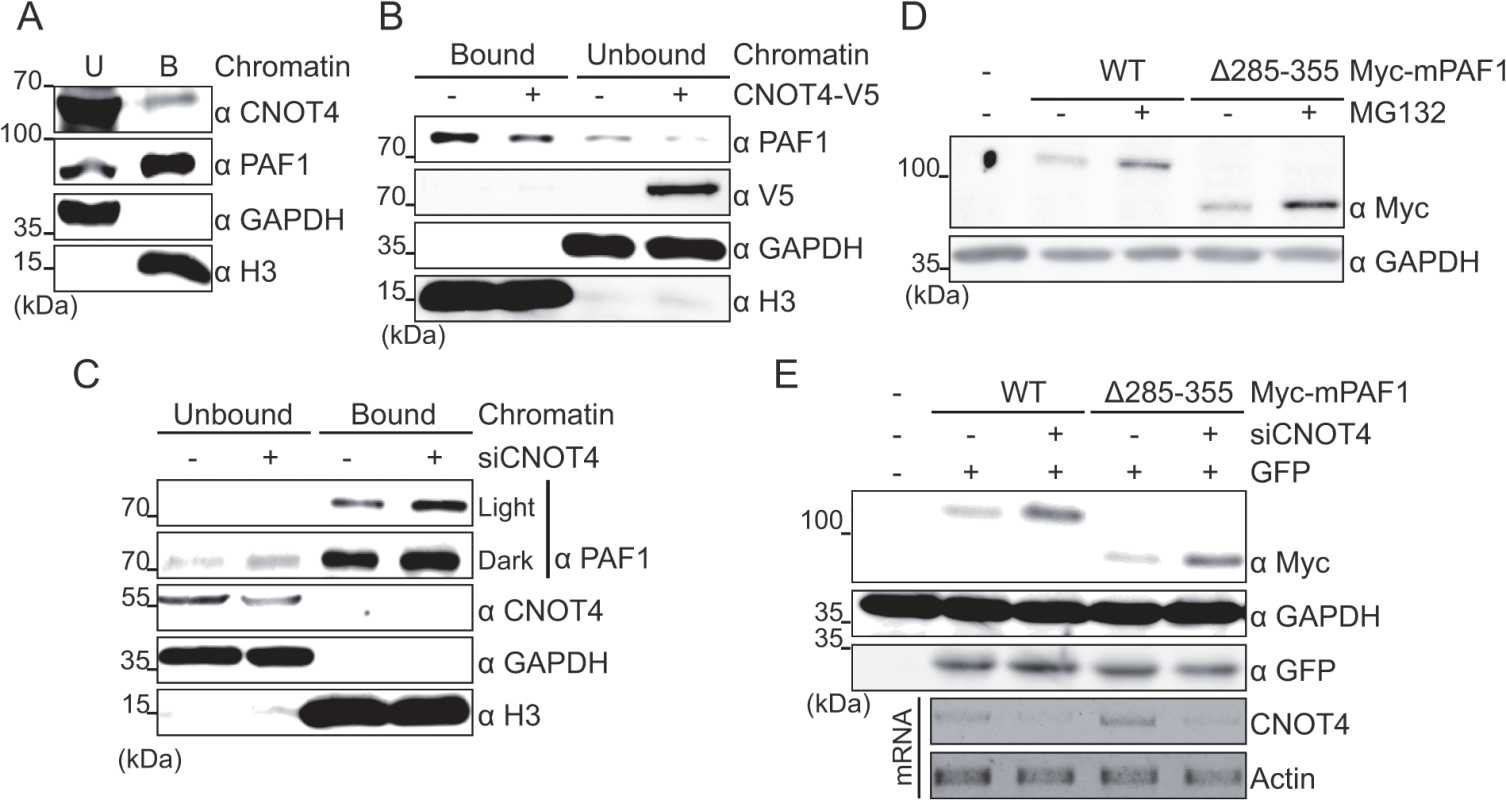
CNOT4 affects PAF1 independent of chromatin binding. (A) HEK293 cells were fractionated into chromatin-unbound (U) and bound (B) fractions, and the chromatin association of the proteins was determined via Western blotting. GAPHD and H3 were used as markers of the chromatin-unbound and bound fractions, respectively. (B, C) Chromatin-unbound and bound PAF1 was determined by immunoblotting in CNOT4-overexpressed (B) and silenced (C) cells. (D) WT or Myc-mPAF1 Δ285-355-transfected HEK293 cells were treated with MG132 (10 μM) for 6 hr, and Myc-tagged PAF1 protein levels in whole-cell lysates were detected. (E) WT or Myc-mPAF1 Δ285–355 was transfected along with control or CNOT4 siRNA. Myc-tagged PAF1 protein levels in whole-cell lysates were determined using an antibody against Myc. The pGFP plasmid was included as a transfection control. CNOT4 depletion was measured by RT-PCR.

The recruitment of PAFc to chromatin and yNot4 were previously shown to be genetically linked in *S*. *cerevisiae* [10]. We therefore examined whether CNOT4 regulates the degradation of PAF1 by controlling PAF1 chromatin association. Both chromatin-unbound and bound fractions were separately prepared and assayed for the presence of PAF1 and CNOT4 (Fig 6A). As reported previously, the majority of PAF1 was found in association with chromatin [22], whereas CNOT4 was mostly present in the chromatin-unbound fraction. When CNOT4 was overexpressed, the levels of endogenous PAF1 protein were reduced in both the chromatin-bound and unbound fractions (Fig 6B). Similarly, depleting CNOT4 resulted in increases PAF1 in both the chromatin-unbound and bound fractions (Fig 6C). These results indicated that unlike yNot4, CNOT4 does not affect chromatin association of PAF1 in our system.

We then used the PAF1Δ285–355 mutant, which does not associate with chromatin but still localizes to the nucleus [22] to test whether chromatin association is required for PAF1 degradation. However, this mutant was degraded normally, and degradation required the 26S proteasome (Fig 6D) and CNOT4 (Fig 6E), indicating that PAF1 chromatin association was not required for the CNOT4- and 26S proteasome-mediated degradation of PAF1 protein.

## Discussion

In the present study, we examined the role of CNOT4 regulating the chromatin association and 26S proteasome-mediated degradation of the PAF1 protein.

We demonstrated that CNOT4 controls the degradation of PAF1 independent of its E3 ligase activity. A CNOT4 mutant that lacks the internal RING domain exhibited similar effects as WT CNOT4 (Fig 1D), and there was no physical interaction between CNOT4 and PAF1. Therefore, PAF1 itself may not be a direct substrate of CNOT4, with the effects of CNOT4 on PAF1 protein degradation instead occurring indirectly. We postulate that the regulation of PAF1 by CNOT4 may occur through one of the following mechanisms. First, CNOT4 may control the expression or activity of unidentified E3 ligases or deubiquitinases that target PAF1 for ubiquitination. Second, as reported for yNOT4, CNOT4 may globally alter ubiquitin and 26S proteasome activity levels, thereby leading to changes in the protein levels of PAF1.

PAF1 is poly-ubiquitinated and degraded via the 26S proteasome. Our data also indicate that PAF1 must be localized to the nucleus in order for it to be degraded by the 26S proteasome. Although CNOT4 was not directly responsible for the ubiquitination of PAF1, it nevertheless influences the ubiquitination status of PAF1. Taken together, these results suggest that CNOT4 might control an early step in the ubiquitination of PAF1, such as nuclear import/export or chromatin association, which functions as a signal for recognition by the E3 ligase.

The dynamic regulation of chromatin association and dissociation by transcriptional complexes is a crucial step controlling transcription. PAF1, along with the PAFc, functions as a critical transcriptional regulator during the entire transcriptional process, with multiple interactions with different proteins at different steps. Although the molecular mechanism underlying the association between chromatin and the PAFc is unclear, accumulated evidence indicates that the modification status of histones affects the chromatin association of the PAF1 protein and, potentially, PAFc. Acetylation of histone H3 disrupts the PAF1-chromatin association [31]. The PAF1 protein directly interacts with H3 or methylated H3 but not with acetylated H3 in vitro, and recruitment of the histone acetyltransferase GCN5 to the target locus blocks PAF1 recruitment [22, 31]. In addition, the overall acetylation levels of histones H3 and H4 are significantly reduced in a yNot4-deleted yeast strain [32].

Therefore, we initially explored the possibility of CNOT4 regulation of PAF1 at the step of chromatin association, particularly as yNot4 is reported to regulate the recruitment of PAFc to chromatin [10]. However, in our cellular system, both the chromatin-unbound and bound form of PAF1 behaved similarly in CNOT4-silenced or overexpressed cells (Fig 6B and 6C), and chromatin association was not required for the CNOT4-mediated degradation of PAF1 (Fig 6E). These results indicate that CNOT4 does not affect the association of PAF1 with chromatin at the global level. Although it is premature to propose a working mechanism, it is highly likely that CNOT4 selectively degrades the chromatin-unbound form of PAF1. However, we cannot exclude the possibility that CNOT4 locally regulates the association of PAFc with chromatin at specific target loci.

Because PAF1 functions as a key protein for the interaction between PAFc and RNA Pol II or elongation factors, we believe that the regulation of PAF1 by CNOT4 plays a key role in the regulation of PAFc-mediated gene expression. The external or internal signals that affect formation of PAFc and the molecular identities that mediate the regulation of its steady-state levels remain to be elucidated.
